# Five Years Follow-Up of Acrysof Cachet® Angle-Supported Phakic Intraocular Lens Implantation for Myopia Correction

**DOI:** 10.1155/2022/5362020

**Published:** 2022-03-26

**Authors:** Aytan Musayeva, Jana C. Riedl, Adrian Gericke, Urs Vossmerbaeumer

**Affiliations:** ^1^Department of Ophthalmology, University Medical Center, Johannes Gutenberg-University Mainz, Langenbeckstr. 1, Mainz 55131, Germany; ^2^Schepens Eye Research Institute, Massachusetts Eye and Ear, Department of Ophthalmology, Harvard Medical School, Boston, MA 02114, USA

## Abstract

**Purpose:**

The Acrysof Cachet® angle-supported phakic intraocular lens (pIOL) (Alcon Laboratories, Inc., Fort Worth, TX) is designed to correct high refractive errors in human eyes. The aim of this study was to evaluate the outcome of AcrySof Cachet® angle-supported pIOL implantation with particular regard to efficacy and safety of the implant over a 60-month follow-up period.

**Design:**

Retrospective consecutive clinical case study.

**Methods:**

Prior to pIOL implantation, patients had a complete ophthalmologic examination including objective and subjective refraction, uncorrected visual acuity (UCVA) and corrected distance visual acuity (CDVA), endothelial cells density (ECD), slit lamp photography, optical coherence tomography (OCT), Scheimpflug digital videokeratoscopy, optical biometry, slit lamp examination, intraocular pressure (IOP) measurement, and pupillometry. Postoperatively, patients received yearly a complete eye examination.

**Results:**

Thirty-one eyes of 16 patients were included in this study. The mean age was 36.2 ± 8.1 years. UCVA (logMAR) improved from 1.33 ± 0.20 before surgery to 0.08 ± 0.14 one year after surgery and was 0.20 ± 0.20 five years after surgery. CDVA (logMAR) improved from 0.10 ± 0.10 before surgery to 0.05 ± 0.13 one year after surgery and was 0.04 ± 0.14 five years postoperatively. The mean percentage of endothelial cells loss (ECL) was 11.51% over the first year and 15.95% five years after surgery. There were no intraoperative complications in any of the eyes.

**Conclusions:**

Our results up to five years after implantation of the AcrySof Cachet® angle-supported pIOL demonstrated very good outcomes in all above shown measurements, including CDVA, UCVA, and ECD. However, since major endothelial cell loss may occur in some patients with this type of pIOL, regular follow-up visits are required.

## 1. Introduction

Phakic intraocular lenses (pIOLs) have been used for more than 60 years for correction of moderate to high ametropia [1, 2]. However, most of the clinically tested models had to be withdrawn from the market due to complications in the medium- or long-term, especially due to loss of corneal endothelial cells and only a few are still used today [3–10]. Two types of pIOLs differing with regard to their level of placement within the eye may be distinguished. First, there are posterior chamber pIOLs, which are placed in the ciliary sulcus, such as the Phakic Refractive Lens (PRL®, Carl Zeiss Meditec, Jena, Germany), no longer available [11–13], and the implantable collamer lens (ICL®, Staar Surgical AG, Nidau, Switzerland) [14]. Second, anterior chamber intraocular lenses are also in use, of which some are fixed in the iris (Artisan®, Artiflex®, Verisyse®, Veriflex®, Ophtec BV, The Netherlands) while other are angle-supported (AcrySof Cachet®, Alcon, Fort Worth, TX, USA), no longer available [15, 16].

The AcrySof Cachet® angle-supported pIOL (Alcon Laboratories) is a single-piece, foldable, soft acrylic lens (acrylate/methacrylate copolymer) with a 6.00 mm diameter optic and with an overall length of 12.5 to 14.0 mm and a dioptric range of −6.00 to –16.50 D in 0.5 D steps [17, 18]. Made from foldable hydrophobic acrylate, the haptics are easily foldable and the IOL can be inserted through a 2.6 mm incision, which reduces the risk of damage to angle structures and pupil ovalization [19]. A good level of refractive stability, predictability, and safety has previously been reported for the pIOL [17, 19–21]. Compared to other surgical possibilities to correct high refractive errors, the implantation of a pIOL has several advantages [22–32]. First, the crystalline lens retains its function, and vitreoretinal complications are less likely to occur [33, 34]. Second, the pIOL is removable, which allows for reversibility of the preoperative condition [35, 36]. Furthermore, refraction is stable and the method is adjustable with complementary corneal refractive procedures [37–39]. Despite many advantages, pIOL surgery has a wide spectrum of potential long-term complications, such as endothelial cell loss, intraocular inflammation, pupil distortion, cataract formation, and secondary glaucoma. Due to concerns about endothelial cells loss in a group of 1323 eyes implanted, Alcon voluntarily discontinued the production of this intraocular lens in 2014. This study aims to critically evaluate the performance of the Cachet® pIOL, notably concerning predictability and stability of the refractive results, the risk profile (safety), and patient satisfaction on a long-term follow-up basis.

## 2. Methods

Thirty-one eyes of 16 patients, who required surgical correction of high myopia to achieve spectacle independence between June 2010 and July 2012 at the Department of Ophthalmology, University Medical Center Mainz, were included in this study. The mean age of the patients was 36.19 ± 8.093 years, with a range from 23 to 50 years. The mean preoperative value of refraction, spherical equivalent, was −9.669 ± 2.730 D (min. −5.500 D, max. −16.000 D). The mean lens power of implanted IOLs was −10.240 ± 2.422 D, ranging from −6.000 to −15.500 D.

The inclusion criteria were age ≥18 years, stable refraction for at least 1 year, endothelial cells density (ECD) >2200 cells/mm^2^, mesopic pupil size <7.5 mm, anterior chamber depth (ACD) >3.0 mm, wish of spectacle independence, and ametropia not sufficiently correctable with excimer laser surgery.

Contraindications for implantation included ACD <3.0 mm, insufficient ECD (<2200/mm^2^), anomaly of the iris or pupil, active infectious disease, recurrent or chronic uveitis, clinically significant cataract, posterior segment pathologies, such as macular degeneration or other macular and retinal abnormalities, and glaucoma.

Prior to pIOL implantation, patients received a complete ophthalmologic evaluation including

  Uncorrected (UCVA) and corrected distance visual Scheimpflug acuity (CDVA)
  Subjective and objective refraction
  Measurement of ECD (SP-3000 P, Topcon, Willich, Germany)

Scheimpflug digital videokeratoscopy (Pentacam®, Oculus Wetzlar, Germany) was performed with the measurement of

(1) Corneal topography and corneal thickness
(2) ACD
(3) Angle-to-angle distance.
    Axial length and ACD measurement (IOL Master (Carl Zeiss Meditec AG, Jena, Germany)
    Slit lamp and a fundus examination (Haag-Streit slit lamp, Bern)
    Measurement of IOP (Goldmann applanation tonometry, Haag-Streit AG, Köniz, Switzerland)
    Pupillometry (Colvard Pupillometer, Glendora, USA)

Postoperative follow-up visits were scheduled at day 1, week 1, and month 1, 3, 6, 12, 24, 36, 48, and 60 including the following examinations at given points of time:

  UCVA and CDVA
  Subjective and objective refraction
  Measurement of ECD
  Anterior segment photography (rotational stability and pupil geometry)

Anterior segment optical coherence tomography (OCT, using OCT Spectralis, Heidelberg Engineering, Heidelberg, Germany) was performed to measure

1. Position of the implant in the anterior chamber;
2. Position of the haptics in the angle;
3. Distance between the posterior surface of the pIOL and the anterior surface of the crystalline lens.

Scheimpflug digital videokeratoscopy was used to measure
  1. Distance between the anterior surface of the pIOL and the central corneal endothelium;
  2. ACD;
Slitlamp assessment and a fundus examination
Measurement of IOP (Goldmann applanation tonometry)
We used a questionnaire developed at our department to assess patient satisfaction after pIOL implantation.

Surgical procedure and postoperative treatment: surgery was performed under topical anesthesia, implanting one pIOL into one eye per session. All surgeries were performed by the same experienced ophthalmic surgeon (UV) following the manufacturer's recommendations. The pIOL was implanted through a 3.2 mm incision using the proprietary shooter system. Pupil constriction was obtained by topical administration of 0.5% pilocarpine eye drops and endothelial protection was ascertained using Viscoat® (Alcon, Ophthalmic Viscosurgical Device). The implantation process followed exactly the scheme as proposed by the manufacturer. Intraoperative viscoelastic removal was performed with bimanual irrigation/aspiration handpieces with low bottle height (infusion pressure approximately 25 mmHg). Complete removal was verified meticulously. Two hours postoperatively, all patients were subject to slit lamp examination and applanation tonometry of intraocular pressure. In all cases, readings were in physiological range without need of surgical revision or application of IOP-lowering medication. Two hours after surgery, the patient was visited to ensure complete removal of the viscoelastic material by IOP measurement.

**Figure 1:**
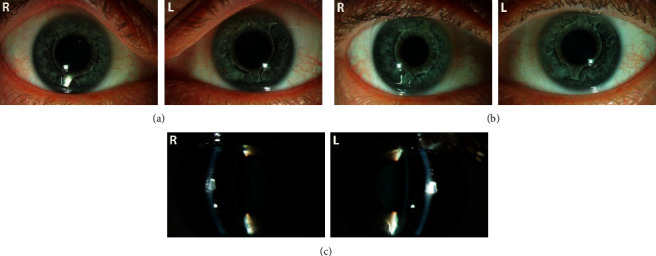
Slit lamp images of the AcrySof Cachet angle-supported pIOL in the anterior chamber postoperatively. (a) 1 year, right (left image column) and left (right image column) eye. (b) Five years postoperatively, in the same patient. Note the slight rotation of the IOL especially in the left eye (right image column). (c) Vertical slit lamp image for control and exclusion of any contact between the pIOL, the cornea, or the crystalline lens.

Postoperative treatment contained of unpreserved ofloxacin eye drops 4 times daily for 5 days and prednisolone eye drops 3 times daily for 3 weeks. The patients received yearly a complete eye examination including the above given measurements and also a photograph to control position, rotational stability, and pupil geometry (Figure 1).

### 2.1. Statistical Analysis

Power calculation was conducted for ECL based on previously published data [40]. For the 31 eyes included in the present study, the power was 82% and 97% for comparison of preoperative with one year postoperative and five years preoperative results, respectively (paired *t*-test and *α* = 0.05). For comparisons of postoperative values with the preoperative value, a paired *t*-test was used. Since each of the five postoperative time points was compared with the preoperative value, a correction for multiple comparisons was made by the Bonferroni correction, which resulted in a reduction of critical *α* to 0.01.

## 3. Results

### 3.1. Visual Acuity

**Figure 2:**
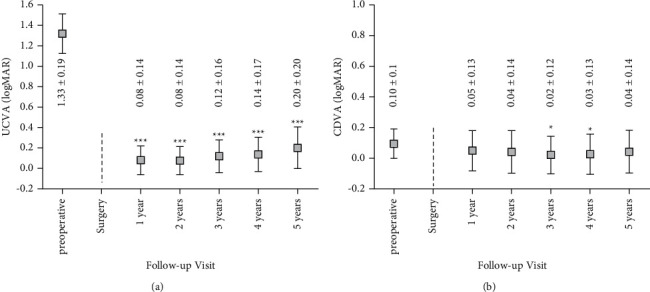
(a) UCVA pre- and up to 5 years after pIOL implantation. Values are presented as mean ± SD (^*∗∗∗*^*p* < 0.0001). (b) CDVA pre- and up to 5 years postoperatively. Values are presented as mean ± SD (^*∗*^*p* < 0.01).

UCVA (logMAR) improved from 1.33 ± 0.19 before surgery to 0.08 ± 0.14 one year after surgery and was 0.20 ± 0.20 five years after surgery (^*∗∗∗*^*p* < 0.0001 for each year compared to preoperative values, Figure 2(a)). CDVA (logMAR) improved from 0.10 ± 0.10 before surgery to 0.05 ± 0.13 one year after surgery and was 0.04 ± 0.14 five years postoperatively. Respective *p* values were *p*=0.0339 (one year after surgery versus preoperative), *p*=0.0293 (two years after surgery versus preoperative), ^*∗*^*p*=0.0014 (three years after surgery versus preoperative), ^*∗*^*p*=0.0085 (four years after surgery versus preoperative), and *p*=0.0322 (five years after surgery versus preoperative) (Figure 2(b)). In none of the patients, a decrease of visual acuity (UCVA and CDVA) has been observed.

### 3.2. ECD

**Figure 3:**
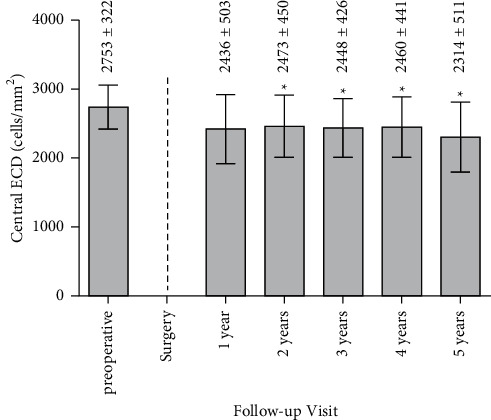
Individual ECD pre- and up to 5 years postoperative. Values are presented as mean ± SD (^*∗*^*p* < 0.01).

ECD was 2753 ± 322 cells/mm^2^ preoperatively, 2436 ± 503 cells/mm^2^ one year postoperatively, and 2314 ± 531 cells/mm^2^ five years postoperatively, which corresponds to an endothelial cell loss (ECL) of 11.51% over the first year and of 15.95% after 5 years (Figure 3). Respective *p* values were *p*=0.0159 (one year after surgery versus preoperative), ^*∗*^*p*=0.0034 (two years after surgery versus preoperative), ^*∗*^*p*=0.0022 (three years after surgery versus preoperative), ^*∗*^*p*=0.0064 (four years after surgery versus preoperative), and ^*∗*^*p*=0.0071 (five years after surgery versus preoperative). The percentage of the eyes with an ECL of 25% or greater of preoperative ECD was 6.45% (2 of 31 eyes) [41]. In all other cases, total ECL was under 25%. The percentage of eyes with final (at the end of the follow-up period) ECD lower than 1500 cells/mm [2] was 3.23% (1 of 31 eyes). The percentage of eyes with postoperative annual ECL (excluding surgical trauma) higher than the expected physiological maximum (i.e., >1.6% loss of annual ECD) [7] was 22.58% (7 of 31 eyes).

### 3.3. IOP

**Figure 4:**
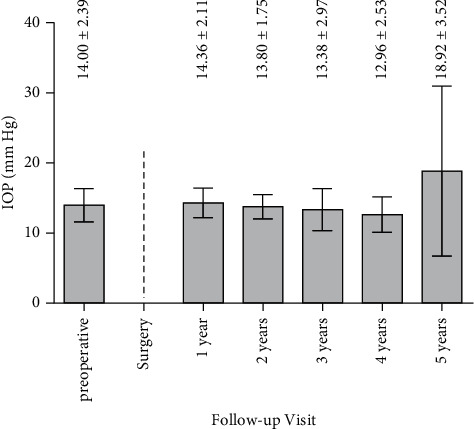
IOP pre- and up to 5 years postoperative.

The mean IOP was 14.00 ± 2.39 mmHg before surgery, 14.36 ± 2.11 mmHg one year after surgery, and 18.92 ± 3.52 mmHg 5 years postoperatively. No marked IOP changes were observed during the whole postoperative period (Figure 4). Respective *p* values were *p*=0.6454 (one year after surgery versus preoperative), *p*=0.2247 (two years after surgery versus preoperative), *p*=0.1509 (three years after surgery versus preoperative), *p*=0.3523 (four years after surgery versus preoperative), and *p*=0.0991 (five years after surgery versus preoperative).

### 3.4. Complications

There were no intraoperative complications in any of the eyes. However, in the first 24 hours postoperatively, two patients had a slightly elevated IOP of 23 mmHg, which dropped back to normal levels without any therapy within the following days. In one patient (43 years old when operated), there was a profound ECL of 63.82% after 5 years. The anterior chamber situation was normal without any inflammation. The distance between the pIOL and the lens as well as the position of the pIOL in the anterior chamber was stable, and the rotation was <5°.

Because of the marked ECL, we decided to explant the Cachet® pIOL with immediate sequential implantation of an ICL®.

Another patient (31 years old when operated), who had undergone Cachet® pIOL implantation in both eyes, experienced an IOP elevation up to 56 mmHg and 35 mmHg on the right and left eye, respectively, in the 5-years follow-up. After topical therapy with timolol/dorzolamide eye drops 2 times daily and clonidine eye drops 3 times daily for both eyes, the IOP values were between 13 mmHg and 20 mmHg in the right eye and between 12 mmHg and 19 mmHg in the left eye. The anterior chamber situation was normal without any inflammation in this patient. Rotation of the Cachet® pIOL in both eyes was <5°. The distance between the pIOL and the lens on the left eye was significantly smaller than on the right eye, but gonioscopy showed an open, normally appearing anterior chamber angle (Schaffer grade 3). Fundoscopic exam did not reveal any glaucomatous cupping of the optic nerve head. The patient is in regular outpatient examination, and the IOP is within normal limits with the therapy reported above. One patient (35 years old when operated), who received Cachet® pIOL bilaterally, was not satisfied with the postoperative UCVA. Therefore, we decided to perform femtosecond-assisted laser in-situ keratomileusis (Femto-LASIK) ten months after pIOL implantation in both eyes. The refraction after pIOL implantation, which needed correction was sph. −0.75 D cyl −0.25 D/ 77° on the right eye and sph. −0.5 D cyl. −0.5 D/ 35° on the left eye. In none of the patients signs of pigmentary dispersion (pigmentary Tyndall or Krukenberg spindle) have been found in the follow-up examinations. Acute postoperative anterior uveitis was not observed. Pupil ovalization was not observed; pupil shape and motility were preserved in all patients. No iris synechiae, transillumination defects, or sphincter erosions were found. No opacities of the crystalline lens or decrease of the crystalline lens transparency could be found in any patient. There was no incidence of retinal detachment.

### 3.5. Patient Satisfaction

Three patients reported on night halos, two patients on increased light sensitivity at night, and one patient on glare 1 year postoperatively. With time, patients seemed to get used to these symptoms and it was not significant at the 5-year follow-up. As part of a satisfaction questionnaire, patients were asked if they would have the same pIOL implanted again, and at the 5-year postoperative visit, this question was positively answered by 94% (*n* = 15 of 16, Table 1).

## 4. Discussion

Our study demonstrated a high efficacy of Cachet® angle-supported pIOL implantation in correction of high refractive errors. We observed an increase in both UCVA and CDVA, which is in line with previous studies [42, 43]. We also compared the results of this study with other reports to point out the reasons of the most common complications.

Damage to the anterior chamber structures, especially to the corneal endothelium by the pIOL is the most specific complication. In a 7-year follow-up study, Alio et al. revealed an ECL of 3.8% in the first year, but of only 0.5% per year after the second postoperative year [44]. Kohnen et al. also reported on ECL in a 5 year follow-up after Cachet® pIOL implantation [20]. Six months after surgery, mean acute reduction in central ECL was 3.3%, but only 1.3% between the 6-month and 5-year visits, respectively. In a recently published long-term analysis with the AcrySof Cachet® angle-supported pIOL, an ECL of greater than 30% from the preoperative baseline at any time after implantation affected 8.0% of all eyes and resulted in pIOL explantation in 3.1% of all eyes [21]. In our study, we found an average ECL of 11.51% between the preoperative measurement and the measurement 1 year postoperatively. During the following years, additional ECL was only minor. ECD was reduced by 15.95% five years after surgery compared to the measurement before surgery, suggesting that the surgical procedure was the main cause of ECL, which is in agreement with previous findings [45].

Pupil ovalization is regarded as a result of excessive continuous pressure upon the anterior chamber angle from the haptics. It is not only a cosmetically disturbing problem, but also causes glare. Alio et al. reported on pupil ovalization in 5.9% of patients, two of whom needed pIOL explantation [44]. Perez-Santonja et al. revealed that most eyes (71.1%) with Cachet® pIOL did not show rotation of more than 15° and it was not clinically relevant in any of the cases [46]. Retinal complications following pIOL implantation in patients with extreme myopic eyes [47, 48] due to vitreous instability caused by the surgery have also been reported in previous studies. In our patient group, there was no case of pupil ovalization or of posterior segment changes, such as retinal detachment. Uveitis was also not observed in our study although it has previously been shown to be one of the complications of pIOL implantation [49]. In addition, other adverse events previously associated with pIOLs, including endophthalmitis, hyphema, hypopyon, pIOL dislocation, or pupillary block were not observed in the present study. The crystalline lens remained clear in all patients. Postoperative elevation of IOP, a known complication of pIOL implantation [50], was seen in only one patient in our study. The IOP was in normal limits with topical therapy, and no glaucomatous cupping of the optic nerve was observed. Of the patients that had received the AcrySof Cachet® angle-supported pIOL in our study, 94% became spectacle independent, and this result remained stable for the whole follow-up period.

In conclusion, the AcrySof Cachet® pIOL showed excellent results in our retrospective study. There was only one case of IOL explantation because of marked ECL, which is of similar extent as in the recently published long-term analysis of Kohnen et al. [21]. Our results up to 5 years after implantation of the AcrySof Cachet® angle-supported pIOL demonstrated very good outcomes in all the above shown measurements, including CDVA, UCVA, ECD, and IOP. As already mentioned above, Alcon decided to discontinue the production of this phakic intraocular lens in 2014 due to ECL. Therefore, regular follow-up visits with regular ECD examinations are necessary for those patients, who had this type of pIOL implanted.

## Figures and Tables

**Table 1:** Results of patient questionnaire.

Questions	Number of patients (*n*)	Rate (%)
Night halos	3	18
Glare	2	12
Light sensitivity	3	18
Vision satisfaction when driving a car	16	100
Twilight vision satisfaction	15	94
Night vision satisfaction	14	88
Patients, who would recommend this pIOL to others	15	94

## Data Availability

The data used to support the findings of this study are available on request.
